# New Role of Protein Misfolding Corrector in the ER Stress-Inflammation Axis: Possible Therapeutic Indication in Neuronal and Epithelial Tumor Cells

**DOI:** 10.3390/ijms262210846

**Published:** 2025-11-08

**Authors:** Michela Pecoraro, Adele Serra, Maria Julia Lamberti, Maria Pascale, Silvia Franceschelli

**Affiliations:** 1 Department of Pharmacy, University of Salerno, Via Giovanni Paolo II, 84084 Fisciano, Italy; adserra@unisa.it (A.S.); pascale@unisa.it (M.P.); 2 Ph.D. Program in Drug Discovery and Development, University of Salerno, 84084 Fisciano, Italy; 3 Instituto de Biotecnología Ambiental y Salud (INBIAS UNRC CONICET)-UNRC, Río Cuarto 5800, Argentina; mlamberti@exa.unrc.edu.ar; 4 Departamento de Biología Molecular, Facultad de Ciencias Exactas, Físico-Químicas y Naturales, Universidad Nacional de Río Cuarto, Río Cuarto 5800, Argentina

**Keywords:** neurodegenerative disease, ER stress, corrector, misfolding protein, inflammation

## Abstract

Protein misfolding diseases are characterized by structurally abnormal proteins that lose their functionality, resulting in cellular and tissue dysfunction. Neurodegenerative diseases, including Parkinson’s disease, Alzheimer’s disease and Huntington’s disease, share a common etiopathogenesis characterize by the accumulation of misfolded proteins. These proteins autonomously aggregate within neuronal cells, triggering inflammation and cell death. The accumulation of misfolded proteins triggers endoplasmic reticulum (ER) stress, leading to alter Ca^2+^ homeostasis. This prolonged stress condition induces the cleavage of procaspase 4 which is resident in ER and activates NF-kB pathway activation, leading to inflammatory responses and cell death. In this study, the efficacy of the drug Vx-445 (Elexacaftor), used in the pharmacological treatment of cystic fibrosis, was assessed in human adenocarcinomic basal alveolar epithelial (A549) and neuronal (SH-SY5Y) cell lines, where ER stress was induced by Thapsigargin. The aim was to assess whether the corrector was able to reduce ER stress by restoring cellular homeostasis and, probably, the proper folding of misfolded proteins and reducing the inflammatory response triggered by these events. Therefore, protein levels of IkBα, p-STAT 3 and COXII were analyzed by flow cytofluorimetry, while Ca^2+^ content was measured by spectrofluorimetry. The results obtained suggest a significant effect of Vx-445 in restoring cellular homeostasis, leading to reduced expression of inflammation-related proteins, such as IL-6, tested by ELISA. Although preliminary, these results encourage further studies to explore the potential repurpose of Vx-445 as a therapeutic candidate for conditions involving ER stress and chronic inflammatory diseases associated with protein misfolding, beyond its current use in cystic fibrosis.

## 1. Introduction

The ER is a vast membrane network spanning from the nuclear envelope to the cell periphery. In mammalian cells, it sustains homeostasis by regulating intracellular Ca^2+^, lipid/cholesterol synthesis, initiation of N-glycosylation essential for proper protein folding, and responses to pro-oxidant stress [1].

Consequently, proteins undergo folding and organization within the ER, while vesicular transport facilitates the direct transportation of non-ER-resident proteins, including secretory or plasma membrane proteins, to the cell surface. When proteins undergo mutation, misfolding, or improper assembly, they become targets for degradation through ER-associated protein degradation (ERAD) or autophagy processes. These pathways involve the complete destruction of entire sections of the ER. If these degradative pathways prove ineffective, signal transduction mechanisms are activated, resulting in programmed cell death (apoptosis) as a protective mechanism or the activation of the unfolded protein response (UPR) to restore homeostasis [1,2].

This response triggers a condition known as ER stress, which initiates a complex cascade of signaling events, including the phosphorylation of BiP/GRP78. Notably, GRP78 serves as a gatekeeper of the UPR in mammalian cells. Under normal conditions, GRP78 binds to transmembrane ER stress sensors such as inositol-requiring 1 (IRE1), activating transcription factor 6 (ATF6), and PKR-like initiation factor 2α kinase (PERK). However, during ER stress, misfolded proteins sequester GRP78, leading to its dissociation from these sensors and facilitating the activation of UPR. Furthermore, UPR activation can also be triggered when GRP78 is downregulated in unstressed mammalian cells, confirming that GRP78 is a negative regulator of the UPR in various models. Furthermore, GRP78 also functions as a calcium-binding protein within the ER [3].

In fact, different forms of stress, such as ischemia/reperfusion and nutrient deprivation, can induce protein misfolding and Ca^2+^ dysregulation [4].

Dysregulated Ca^2+^ signaling has been observed in patients with major protein misfolding/unfolding disorders, including Gaucher’s disease, neurodegenerative disease, cystic fibrosis and ischemia-induced neuronal damage [5]. As a pivotal second messenger, Ca^2+^ participates in numerous homeostatic pathways; unlike the cytosol (~50–100 nM), the ER functions as a dynamic Ca^2+^ store (~0.2–2 mM) [6].

Aggregation of misfolded/unfolded proteins can induce ER stress leading to subsequent increase in cytoplasmic Ca^2+^ [7]. Notably, in prion protein infection, the release of Ca^2+^ from ER-resident Ca^2+^ channels is hypothesized to be one of the initial adaptive reactions [8]. Several studies have shown that adaptive signaling related to UPR is inhibited in the SH-SY5Y cell model by disrupting activating transcription factor 6 (ATF6) [9,10]. ER stress-induced activation of pathways mediated by misfolded/unfolded aggregated protein disrupts Ca^2+^ homeostasis and alters the activity of kinases and phosphatases involved in cell cycle regulation and life cellular processes [5,9].

Ultimately, Ca^2+^ disequilibrium and excessive UPR activation can induce apoptosis via ER-resident caspases or Ca^2+^-overload-mediated mitochondrial dysfunction [11].

Evidence suggests that ER stress-induced apoptosis is mediated by either caspase 12 (in mice) or caspase 4 (in humans) [11]. The caspase 4 enzyme is mainly situated in the ER and is closely associated with key proteins involved within the ER and closely associates with crucial proteins implicated in ER stress-induced cell death pathways, including GRP78, a well-known ER stress indicator [12]. Therefore, caspase 4 plays a pivotal role in the pathophysiology of Alzheimer disease and may represent a potential therapeutic target [13].

Additionally, human caspase 4 is classified as an “inflammatory caspase”, which plays a role in the innate immune response [14]. ER stress can both drive and result from chronic inflammation, which accompanies disorders such as diabetes, obesity, neurodegenerative and neuromuscular diseases, arthritis and spondyloarthropathies, respiratory inflammations, and inflammatory bowel diseases; these are often linked to protein misfolding and ER dysfunction, and UPR activation is common in many chronic inflammatory and autoimmune conditions [15]. The three arms of the canonical UPR interact with several inflammation and stress signaling pathways, including the NF-κB-IκB kinase (IKK) and JNK-AP1 pathways, as well as oxidative stress-activated networks, that affect metabolism. ER stress-induced activation of the JAK1/STAT3 axis promotes the expression of IL-6 and several chemokines that drive inflammation [16]. Consequently, inflammation and ER stress are intricately interconnected at multiple levels. Both functions serve as short-term adaptive systems, essential for the organism’s functionality and survival. However, when chronically engaged, both inflammation and ER stress can become detrimental [17].

Recent studies conducted by our research group [18,19] have shown that Vx-809 (Lumacaftor), a widely prescribed drug for cystic fibrosis (CF), not only enhances Cystic Fibrosis Transmembrane Conductance Regulator (CFTR) proteostasis but also exerts broader protective effects in non-CF models, reducing ER stress markers, restores intracellular Ca^2+^ homeostasis, and mitigates inflammation in A549 pulmonary epithelial cells, which are frequently used as a consistent pattern for human type II alveolar lung epithelium and have been broadly utilized for the study of pharmacology, toxicology, lung lesion and metabolic processes of lung tissue in vitro [20]. Additionally, it showed potential applicability in other protein misfolding-associated disorders by attenuating inflammation in SH-SY5Y neuroblastoma cells. Vx-445, a CFTR modulator that facilitates the processing and trafficking of CFTR was approved as part of a triple combination therapy (with Tezacaftor and Ivacaftor) for CF therapy. It binds to the NBD1 domain of CFTR, thereby enhancing protein maturation and stability [21]. However, its potential off-target effects on other misfolded proteins or ER stress pathways remain unexplored.

In this study, we propose that Vx-445 may act as a broader modulator of proteostasis by mitigating ER stress and its downstream consequences, including inflammation, in non-CF cellular contexts. To evaluate this hypothesis, we induced ER stress using Thapsigargin (TG) and assessed the effects of Vx-445 on key cellular processes. This strategy enabled us to investigate the potential of Vx-445 to modulate ER stress and inflammation beyond its well-established role in cystic fibrosis treatment.

## 2. Results

### 2.1. Vx445 Corrector Counteracts Calcium Homeostasis Dysregulation in ER in Response to Stress

**Figure 1:**
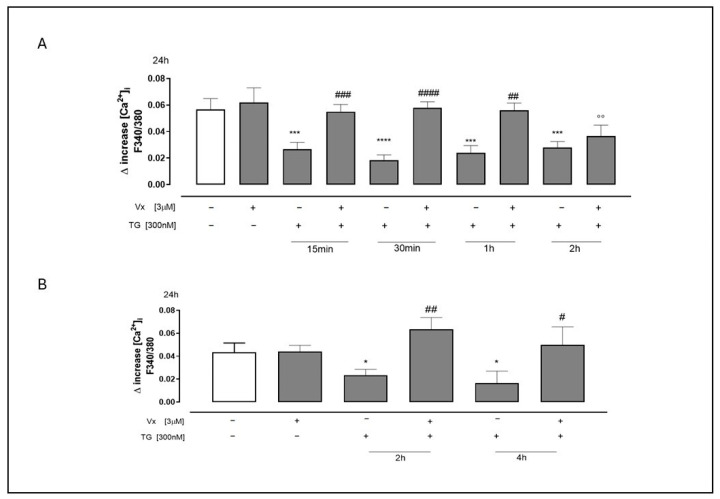
Vx445 counteracts dysregulation of calcium homeostasis. SH-SY5Y (**A**) and A549 (**B**) cells were pretreated with 300 nM TG for 15 or 30 min, 1 or 2 h, and 2 or 4 h, respectively, to induce ER stress. Vx-445 (3 µM) was then added for 24 h. The impact of Vx-445 on the reticular calcium content was assessed in calcium-free media with 1 nM TG present after ER stress induction. The statistics show the average ± standard error of the delta (Δ) increase in Fura 2 proportion—340/380 nm—fluorescence from at least three separate, duplicate tests. The data was analyzed using the Mann–Whitney U test. * *p* < 0.05, *** *p* < 0.001 and **** *p* < 0.0001 compared to untreated cells; °° *p* < 0.005 compared to cells treated with Vx-445; # *p* < 0.05, ## *p* < 0.005, ### *p* < 0.001, and #### *p* < 0.0001 compared to cells treated with TG. The first white column of the bar chart represents the untreated cells.

Given the established link between ER stress and the development of various diseases resulting from protein misfolding, as well as the dysregulation of Ca^2+^ concentration within the ER [22], we used Fura 2-AM to study the effects of Vx-445 administration on intracellular calcium concentrations in a calcium-free incubation medium. The reticular calcium content was assessed using TG (1 nM). As shown in Figure 1, Vx-445-treated cells that had been pretreated with TG exhibited a significantly greater D increase in reticular calcium levels compared to TG-treated cells. Specifically, this effect was observed at 15–30 min and at 1–2 h in SH-SY5Y cells (A) and at 2–4 h for A549 cells (B) (*p* < 0.0001). This finding suggests that in the presence of Vx-445, the ER stores more calcium, thereby improving calcium homeostasis.

### 2.2. The Corrector Interferes in the Cleavage of Procaspase 4

**Figure 2:**
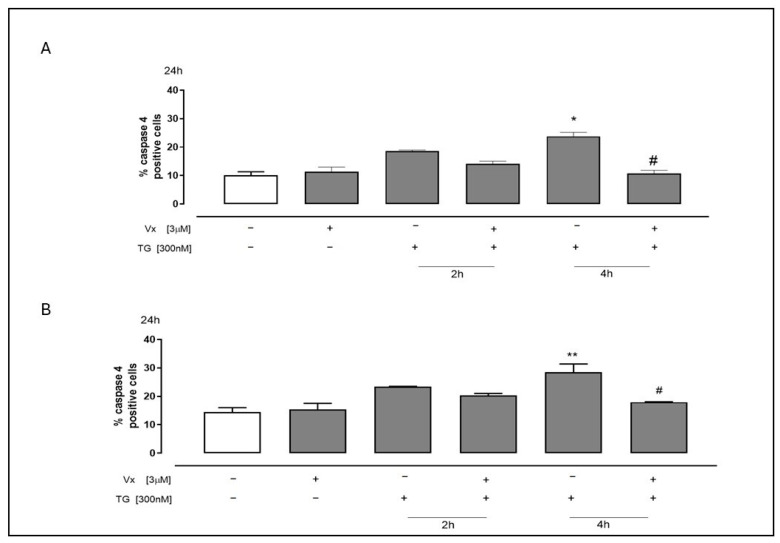
The corrector interferes with the cleavage of procaspase 4 induced by ER stress. To create ER stress, 300 nM TG was administered prior to treatment for 2 or 4 h in SH-SY5Y (**A**) and A549 (**B**) cells. After that, Vx-445 (3 µM) was given for 24 h. The mean ± S.E.M. was used to determine the proportion of caspase 4-positive cells from at least three different experiments, each conducted in triplicate. The data was analyzed using the Mann–Whitney U test. # *p* < 0.05 versus cells treated with TG; ** *p* < 0.005 and * *p* < 0.05 versus untreated cells. The first white column of the bar chart represents the untreated cells.

The accumulation of unfolded and misfolded proteins and the impairment of calcium homeostasis lead to ER stress, initiating a UPR that results in inhibition of protein synthesis, increased expression of ER protein folding chaperones, and/or apoptosis. Caspase 4 is involved in UPR-dependent apoptosis by initiating a series of caspase activations [23]. Consequently, we examined caspase 4 activation in both cell lines treated with Vx-445 under ER stress conditions. Flow cytometric assay showed a significant decrease in levels of caspase 4 (*p* < 0.05), particularly 4 h post treatment (Figure 2). This suggests that the corrector enhances reticular integrity, potentially mitigating ER stress-induced apoptotic signaling, and contributing to its overall cytoprotective effect.

### 2.3. Vx-445 Reduces GRP78/BiP Overexpression Induced by ER Stress

Under non-stressful conditions, the chaperone of the 78 kDa glucose-regulating protein (GRP78/BiP) establishes a link with three UPR sensors, thereby preventing their activation. However, under ER stress conditions, BiP demonstrates an elevated propensity for misfolded proteins, resulting in its dissociation from the UPR sensors. This dissociation facilitates their activation and subsequent downstream signaling pathway [24].

Western blotting analysis of SH-SY5Y and A549 cell lysates showed that TG treatment significantly (*p* < 0.001) increases the expression of BiP in all pretreatment time points, thereby confirming the establishment of ER stress.

**Figure 3:**
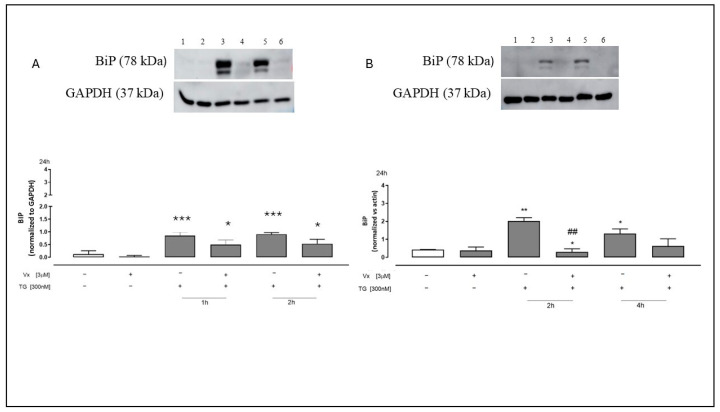
Vx-445 disturbs BiP signaling. Western blot assay: Panel (**A**) (1: non-treated cells, 2: Vx-445 only, 3: TG for 1 h, 4: Vx-445 following a 1 h TG pretreatment, 5: TG for 2 h, and 6: Vx-445 following a 2 h TG pretreatment). Panel (**B**) (1: non-treated cells, 2: Vx-445 only, 3: TG for 2 h, 4: Vx-445 following a 2 h TG pretreatment, 5: TG for 4 h, and 6: Vx-445 following a 4 h TG pretreatment). To create ER stress, both cell types were treated beforehand with 300 nM TG for 1, 2, or 4 h. After that, 3 µM Vx-445 was given for 24 h. Western blot assay was used to evaluate the expression of BiP on neuroblastoma (**A**) and adenocarcinomic (**B**) cells. The expression of the GAPDH protein served as a loading control. The mean ± S.E.M. of at least three separate experiments is used to express the results. To perform statistical analysis, the Mann–Whitney U test was used. The following p-values were used to evaluate significance: * *p* < 0.05, ** *p* < 0.005, and *** *p* < 0.001 for differences compared to cells that were not treated; ## *p* < 0.005 for differences compared to cells that were treated with TG. The first white column of the bar chart represents the untreated cells.

As illustrated in Figure 3, Vx-445 administration reduced TG-induced Grp78/BiP overexpression, suggesting that this corrector may mitigate UPR activation by restoring ER homeostasis.

### 2.4. Vx-445 Attenuates Inflammatory Signaling Through NF-kB and STAT3 Pathways

The cytoplasmic localization of NF-kB is controlled by a family of inhibitory proteins, the IkBs, which bind NF-kB, masking its nuclear localization signal, thus preventing nuclear translocation. Upon exposure to various extracellular stimuli, IkB undergoes rapid phosphorylation, ubiquitination, and ultimately proteolytic degradation, leading to NF-kB releases, nuclear translocation, and activation of gene transcription [25].

Because IkBα protein is a master regulator of NF-kB signaling [26] and is involved in chronic inflammatory processes [27], we chose to examine its expression.

**Figure 4:**
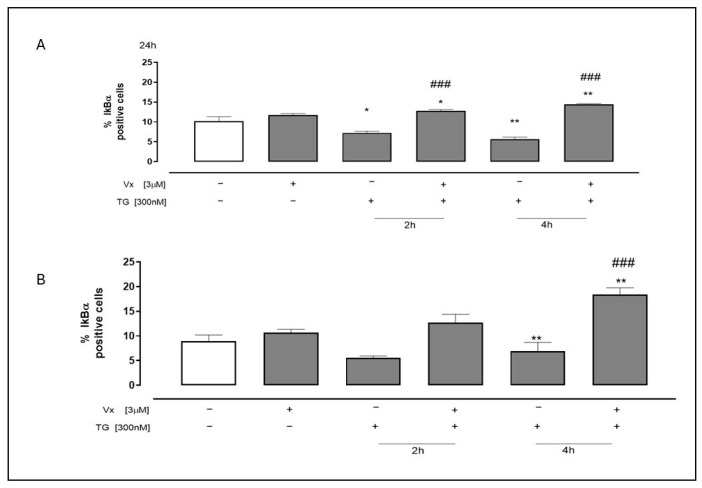
The effect of Vx-445 on the IkBα pathway. To induce ER stress, 300 nM TG was used to pretreat neuroblastoma (**A**) and adenocarcinomic (**B**) cells for 2 or 4 h. Subsequently, 3 µM Vx-445 was administered for 24 h. IkBα levels were assessed by flow cytometry. Data are expressed as mean ± S.E.M. of percentage of IkBα positive cells from at least three independent experiments, each performed in triplicate. Data was subjected to a Mann–Whitney U test analysis. * *p* < 0.05 and ** *p* < 0.005 in comparison to untreated cells; ### *p* < 0.001 compared to cells that were treated with TG. The first white column of the bar chart represents the untreated cells.

As shown in Figure 4, a significant increase in IkBα levels (*p* < 0.0001) was observed using flow cytometry in both Vx-445 treated cell lines under ER stress conditions. These data suggest that Vx-445 treatment shifts the balance towards the non-phosphorylated form of IkBα, potentially leading to NF-kB inhibition.

STAT3 is the most extensively studied anti-inflammatory response signaling pathway responsible for regulating the intensity and duration of inflammation. Its impairment can lead to uncontrolled and increasing inflammation. Membrane receptor signaling mediated by diverse ligands induce JAK kinase activation, subsequently leading to tyrosine phosphorylation of different STAT transcription factors [28].

**Figure 5:**
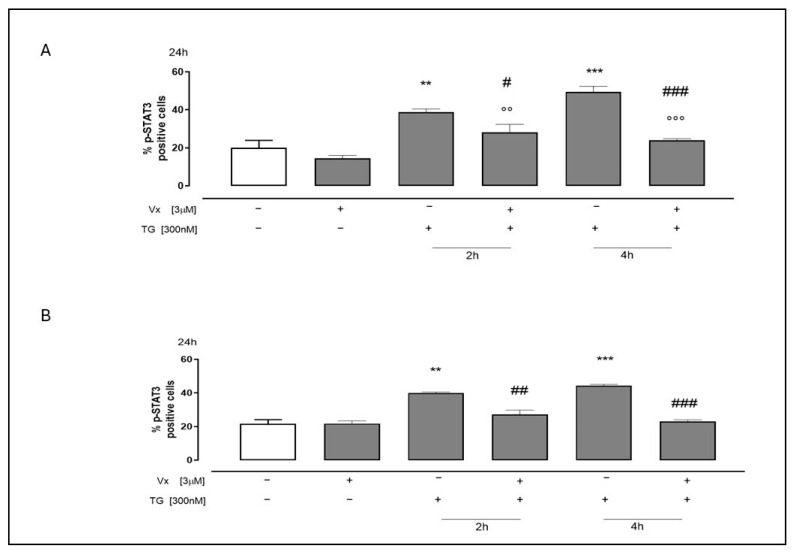
The role of Vx-445 in regulating p-STAT3. Both cell types (SH-SY5Y (**A**) and A549 (**B**)) were pretreated with 300 nM TG for 2 h or 4 h to cause ER stress. Next, 3 µM Vx-445 was added for 24 h. p-STAT3 level were assessed using flow cytometry analysis. Data are expressed as the mean ± S.E.M. of percentage of p-STAT3 positive cells from at least three independent experiments, each performed in triplicate. The Mann–Whitney U test was used to analyze the data. ** *p* < 0.005 and *** *p* < 0.001 versus nontreated cells; °° *p* < 0.005 and °°° *p* < 0.001 versus cells treated with Vx-445; # *p* < 0.05, ## *p* < 0.005 and ### *p* < 0.001 versus cells treated with TG. The first white column of the bar chart represents the untreated cells.

To test STAT3 activation, we analyzed the phosphorylated form of STAT3 (p-STAT3) using flow cytometry in A549 and SH-SY5Y cell lines. The results showed a significant increase (*p* < 0.001) in p-STAT3 levels in cells pretreated with TG for 2 and 4 h compared to untreated cells, confirming the onset of ER stress in our experiments. Vx-445 treatment significantly (*p* < 0.001) decreased the levels of TG-induced p-STAT3, further substantiating its role in modulating the stress response (Figure 5).

Some evidence suggests that NF-κB is involved in gene regulation, neuronal survival, inflammatory responses and cancer [29,30]. Furthermore, NF-κB is a key transcription factor that promotes the induction of pro-inflammatory factors, such as IL-6 [31] and the COXII protein, which, together with STAT3 phosphorylation, contributes to a pro-inflammatory state. We therefore decided to study the expression of IL-6 and COXII in our experimental model.

**Figure 6:**
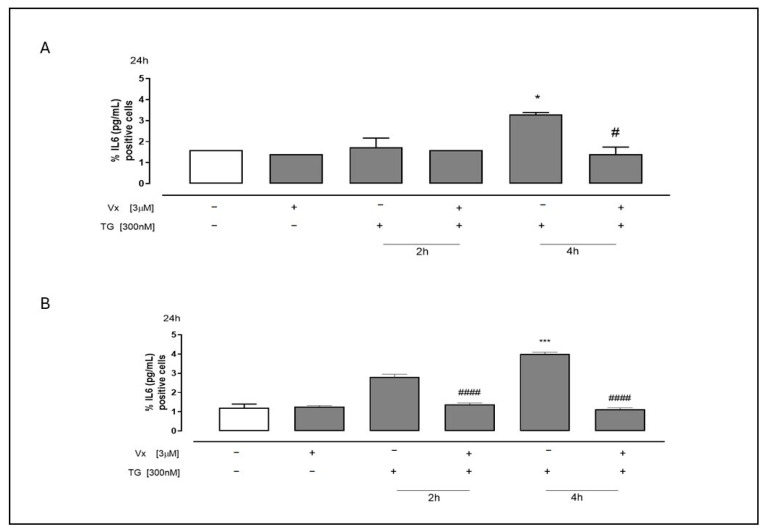
Vx-445 interferes with cytokines expression. The levels of IL-6 in cell supernatant were measured using commercially available ELISA. Both cell types (SH-SY5Y (**A**) and A549 (**B**)) were pretreated with 300 nM TG until 2 h or 4 h to cause ER stress. Next, 3 µM Vx-445 was added for 24 h. The findings are shown as the mean ± standard error of at least three separate, duplicate-run trials. The Mann–Whitney U test was used to evaluate the data. Compared to untreated cells, * *p* < 0.05 and *** *p* < 0.001; instead # *p* < 0.05 and #### *p* < 0.0001 compared to cells treated with TG. The first white column of the bar chart represents the untreated cells.

As shown in Figure 6, ELISA demonstrated a significant reduction (*p* < 0.05) in IL-6 release in Vx-445-treated SH-SY5Y (Figure 6A) and A549 (Figure 6B) cells following TG pretreatment, particularly at 4 h.

**Figure 7:**
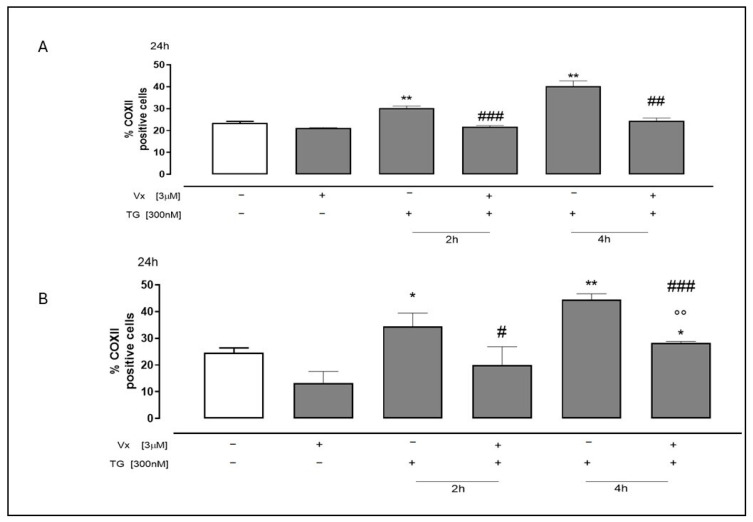
The effect of Vx-445 on COXII levels. To cause ER stress, SH-SY5Y (**A**) and A549 (**B**) cells were pretreated with 300 nM TG for 2 or 4 h. Then, over 24 h, 3 µM of Vx-445 was added. Flow cytometry analysis was used to check the COXII level. The percentage of COXII-positive cells from at least three separate experiments, each conducted in triplicate, was expressed as mean ± S.E.M. The data was subjected to a Mann–Whitney U test analysis. * *p* < 0.05 and ** *p* < 0.005 compared to nontreated cells; °° *p* < 0.005 versus cells treated with Vx-445; # *p* < 0.05, ## *p* < 0.005 and ### *p* < 0.001 compared to cells treated with TG. The first white column of the bar chart represents the untreated cells.

Furthermore, Vx-445 demonstrated the ability to decrease COXII protein expression (Figure 7), as assessed by flow cytofluorimetry, in both cell lines pretreated with TG at both selected experimental time intervals.

These findings suggest that Vx-445 exerts a multi-target anti-inflammatory effect, acting on both upstream signaling molecules (IκBα, p-STAT3) and downstream effectors (IL-6, COXII), highlighting its potential as a therapeutic agent in ER stress-related pathologies.

### 2.5. Vx-445 Interferes in NO Release Under ER Stress

**Figure 8:**
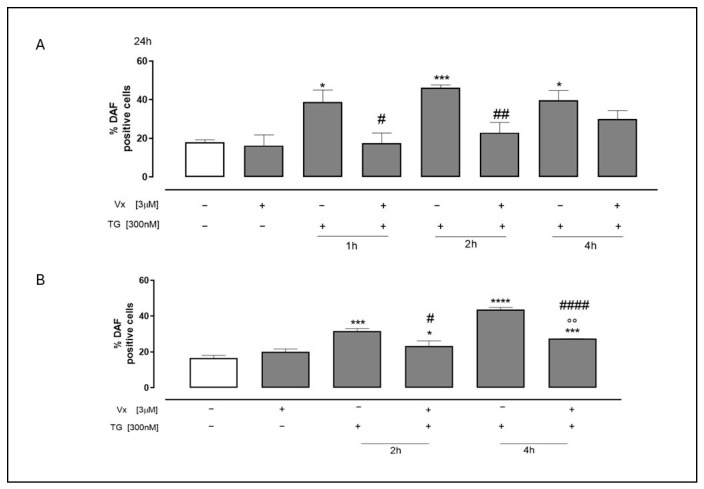
Vx-445 counteracts NO production. To induce ER stress, both cell types were pretreated with 300 nM TG for 1, 2 or 4 h for SH-SY5Y (**A**) and for 2 or 4 h for A549 (**B**). Subsequently, 3 µM of Vx-445 was then added for the entire day. Probe 4,5-Diaminofluorescein Diacetate, DAF-2 DA, has been employed to quantify NO levels. Mean ± SEM of NO generation was expressed as the proportion of DAF-positive cells derived from a minimum of three separate experiments, each carried out in duplicate. The data was assessed using the Mann–Whitney U test. * *p* < 0.05, *** *p* < 0.001 and **** *p* < 0.001 versus nontreated cells; °° *p* < 0.005 versus cells treated with Vx-445; # *p* < 0.05 ## *p* < 0.005 and #### *p* < 0.0001 versus cells treated with TG. The first white column of the bar chart represents the untreated cells.

One signaling molecule that is essential to the pathophysiology of inflammation is nitric oxide (NO). Under typical physiological circumstances, it has an anti-inflammatory action. However, under pathological conditions, excessive NO production acts as a pro-inflammatory mediator, contributing to inflammation [32]. To investigate the effects of Vx-445 on TG-induced NO production, flow cytometric analyses were performed using the fluorescent probe DAF-2DA. Our experiments demonstrated that treatment with Vx-445 significantly (*p* < 0.0001) reduced NO production (Figure 8) in TG-treated cells at all time points. These findings suggest that Vx-445 may reduce ER stress-induced inflammation not only by modulating protein-folding sensors and inflammatory cytokines, but also by limiting excessive NO production.

## 3. Discussion

Protein misfolding is a pathological hallmark of various diseases, including some neurodegenerative disorders, metabolic syndromes, cystic fibrosis, and ischemia [33]. The accumulation of misfolded proteins can lead to the onset of ER stress, resulting in the activation of the UPR, a pathway that plays a key role in reducing such stress. However, under chronic stress conditions, UPR activation can induce inflammation, primarily through the NF-kB pathway. This activation leads to increased production and release of pro-inflammatory molecules. Thus, protein misfolding, ER stress, and inflammation are closely associated with neurodegenerative diseases [34].

Vx-445 (Elexacaftor) is a widely utilized pharmacological agent in the treatment of cystic fibrosis, approved by the FDA in 2019 in combination with the corrector Tezacaftor (Vx-661) and the potentiator Ivacaftor (Vx-770). Although its primary function is to enhance the folding of CFTR protein, the broader role of VX-445 in mitigating ER stress and modulating inflammation remains unexplored.

Therefore, the objective of this study was to ascertain whether Vx-445 can exert its effects on proteins beyond CFTR by reducing ER stress, reversing the activation of the UPR, restoring the folding of misfolded proteins, and thereby reducing the inflammatory process triggered by these events.

To simulate ER stress, cells were pretreated with TG, a non-competitive inhibitor of SERCA (Sarco/Reticular Ca^2+^-dependent ATPase) pump. TG causes Ca^2+^ leakage from the ER, resulting in accumulation of this ion at the cytoplasmic level. This accumulation leads to the accumulation of misfolded proteins, ER stress, UPR activation, and the establishment of stress condition, leading to the activation of the inflammatory response.

Given that the accumulation of misfolded proteins is a contributing factor to numerous diseases, including cystic fibrosis (CFTR), Parkinson’s disease (a-synuclein), and Alzheimer’s disease (β-amyloid), we utilized A549 (alveolar basal epithelial) and SH-SY5Y (neuroblastoma) cells as models.

Protein aggregates are responsible for disrupting Ca^2+^ homeostasis by inducing ER stress. This stress subsequently activates the NF-kB pathway, leading to the activation of the inflammatory process and cell death.

Our spectrofluorimetric analysis demonstrated that VX-445 treatment restored intracellular calcium balance, thereby mitigating cytosolic calcium accumulation. This observation suggests that corrected protein folding alleviates ER stress, thereby preventing calcium dysregulation, which is a trigger for apoptosis and inflammation.

When cells are exposed to ER stress-inducing chemicals, caspase 4, which is confined to the ER membrane, is cleaved; other apoptotic agents do not have this effect. Overexpression of Bcl-2, which prevents signal transduction to mitochondria, had no effect on caspase 4 cleavage, suggesting that caspase 4 is mainly active in ER stress-induced apoptosis [13].

Cytofluorimetric assay demonstrated a reduction in levels of caspase 4 in our experiments due to the decrease in ER distress. This finding corroborates the role of Vx-445 in protein folding. Western blotting analysis further supports this hypothesis by confirming a substantial decrease in GRP78/BiP levels following VX-445 treatment, suggesting an establishment of ER homeostasis. The GRP78/BiP protein serves as a master regulator of the UPR, playing a crucial role in stress adaptation, anti-inflammatory regulation, and immunomodulatory functions [35]. The observed decrease in GRP78/BIP expression indicates that Vx-455 suppresses the UPR, potentially mitigating chronic stress-induced inflammation. ER stress is a key factor in inflammation through the activation of NF-kB and STA3. Our experimental model, demonstrates that treatment with Vx-445 effectively inhibits NF-kB activation, as shown by flow cytometric analysis of Ik-Bα, p-STAT3, NO, and COXII, an enzyme whose induction stimulates the production of inflammatory mediators. Indeed, there is a significant increase in the levels of Ik-Bα, a protein that physiologically maintains NF-kB in its inactive state, resulting in inhibition of the transduction pathway mediated by it, and thus the inflammatory process. In addition, the ELISA showed a significant reduction in the levels of IL-6, a pro-inflammatory cytokine whose expression is regulated by the NF-kB pathway, and in the levels of p-STAT 3, a transcription factor that promotes the activation of pro-inflammatory molecules at the nuclear level. Finally, our experiments showed that VX-455 significantly reduces nitric oxide (NO) production, which further supports the hypothesis that Vx-445 decreases inflammation through pathways including NF-kB, STAT3, and NO signaling.

Our findings with Vx-445 are consistent with previous studies conducted by our group investigating the effects of Vx-809 on non-CF models. In A549 cells, Vx-809 demonstrated a reduction in ER stress markers and modulation of inflammatory responses triggered by TG [18]. Similarly, in SH-SY5Y cells, comparable protective effects were observed, including restoration of calcium homeostasis and reduction in apoptotic signaling [19]. Despite slight variations in experimental conditions, notably the concentrations of correctors employed, the results across all studies collectively reveal a consistent pattern: both Vx-809 and Vx-445 effectively mitigate TG-induced ER stress and subsequent inflammation. These findings substantiate the broader potential of CFTR correctors as modulators of proteostasis and inflammation beyond the context of cystic fibrosis. It is important to emphasize that, although we observed multiple biological effects associated with Vx-445 treatment, our analysis does not provide direct experimental evidence that all these effects are part of a single signaling pathway. Therefore, in the future, more targeted studies are needed to validate the causal connection between the different observed phenomena and to precisely define the molecular pathways involved.

## 4. Materials and Methods

### 4.1. Reagents

The corrector Vx-445 (S8851) was obtained from Selleckchem, (Houston, TX, USA). Monoclonal antibodies utilized are anti-Grp78/BiP (Cell Signaling, 3177, Danvers, MA, USA), anti-COXII (Santa Cruz Biotechnology, sc-19999), anti-p-STAT3 (Santa Cruz Biotechnology, sc-8059), anti-IkB-α (Santa Cruz Biotechnology, sc-371), anti-caspase 4 (Santa Cruz Biotechnology, sc-1229), and anti-GAPDH (Santa Cruz Biotechnology, sc-32233). The secondary antibody (anti-rabbit, A120-101P) was provided by Bethyl Laboratories (Montgomery, TX, USA). Human IL-6 uncoated ELISA kit (88-7066-88, Invitrogen, Carlsbad, CA, USA) and Texas red-conjugated secondary antibody (T6390) were purchased from Thermo Fisher Scientific (Waltham, MA, USA). Fura-2AM (47989), DAF-2DA (251505-M) and Thapsigargin (TG, 586006) were acquired from Sigma-Aldrich (St. Louis, MO, USA).

### 4.2. Cell Culture

Human neuroblastoma cell lines (SH-SY5Y, CL0208, Elabscience, Houston, TX, USA) and human adenocarcinomic alveolar basal epithelial (A549, CL0016, Elabscience) were cultured in 75-cm^2^ sterile flasks with Dulbecco’s modified Eagle’s Medium (DMEM; Euroclone, Pero, Milano, Italy) and DMEM/F12 (Euroclone), respectively, with 10% non-heat inactivated fetal bovine serum (FBS; Euroclone), 100 μg/mL streptomycin, 100 U/mL penicillin, and 2 mM of glutamine, in a humidified atmosphere of 5% CO_2_, at 37 °C. Cells with confluence less than 80% were used.

### 4.3. Experimental Protocol

Cells were seeded and allowed to adhere for 24 h. To induce ER-stress, SH-SY5Y cells were pretreated with Thapsigargin (TG 300 nM) for 1–2–4 h, while A549 cells were treated for 2–4 h. TG primarily inhibits the ER calcium pump, depleting ER calcium stores and thereby disrupting the protein folding process. This leads to accumulation of unfolded proteins within the ER, triggering ER stress [36].

Following the removal of TG, fresh medium was completed to cells with and without Vx-445 corrector (3 µM) for 24 h. Cells were subsequently collected 24 h post treatment for both Vx- and TG-treated cells.

### 4.4. Protein Extraction and Western Blot Analysis

Both cell (7 × 10^5^ cells/plate) lines were plated in 100 mm diameter tissue culture plates and treated after 24 h, as previously described. Proteins were extracted by freezing and thawing the cells in RIPA buffer (150 mM NaCl, IGEPAL 1%, 1 mm EDTA, protease inhibitor cocktail and K^+^ Hepes pH 7.5 20 mM). Lysates were subsequently centrifuged for 15 min at 14,000 rpm (4 °C). The Bradford test was used to determine the protein content, and 30 µg of protein was loaded onto 10% acrylamide gel and then separated via SDS-PAGE in denaturing conditions. After that, the cells were transferred on nitrocellulose membranes (Bio-Rad Laboratories, Richmond, BC, Canada) using a minigel device (Bio-Rad Laboratories, Richmond, BC, Canada). After being blocked for one hour at room temperature in Tris-buffered saline with 5% nonfat dry milk, the membranes were incubated with primary antibodies at 4 °C overnight. GAPDH was employed like loading control. Subsequently, PBS with 0.1% Tween was employed for washing, and secondary antibody was included. Reagents for enhanced chemiluminescence (ECL) and blot imaging (LAS 4000; GE Healthcare, Milano, Italy) were used to visualize the immunoreactive protein bands. ImageJ software (version number 1.44) was employed to quantify the Western blot data.

### 4.5. Flow Cytometry Assay

Protein expressions (anti-caspase 4, COXII, pSTAT3 and ikBα) were analyzed by fluorescence-activated cell sorting (FACSscan; Becton-Dickinson, Franklin Lakes, NJ, USA). Both cell lines were grown on a 12-well plate (each well was seeded with 5 × 10^4^ cells) allowed to adhere for 24 h, and then treated as described above. Next, cells were mechanically harvested and fixed in a solution containing 4% formaldehyde and PBS for 15 min. After washing, cells were blocked with 2% BSA and PBS with 0.1% sodium azide for another 20 min Then, cells were permeabilized with buffer containing Triton X-0.1%, 2% BSA, and PBS in the presence of 0.1% sodium azide for 30 min. Subsequently, the cells were incubated with primary antibodies, followed by Texas-Red conjugated secondary antibodies. Finally, after being rinsed with fixation buffer, cells were detected using flow cytometry and examined using Cell Quest software (version 4.1). The values were reported as the percentage of positive cells for caspase 4.

### 4.6. Analysis of Reticular Calcium Homeostasis

Intracellular calcium levels were quantified using Fura 2-AM, a fluorescent indicator dye. Shortly, cells (3 × 10^4^ cells/plate) were seeded in 6-well culture plates and treated as described in the experimental protocol. Next, after being rinsed in PBS, cells were suspended for 45 min in Hank’s balanced salt solution (HBSS) containing 5 μM Fura 2-AM. Following incubation, cells were rinsed to eliminate extra Fura 2-AM and then incubated for 15 min in calcium-free HBSS/0.5 mM EGTA solution to allow Fura 2-AM to hydrolyze into Fura 2, its active dye form. Cells were then transferred to a spectrofluorimeter (LS-55, Perkin-Elmer, Milano, Italy). Thapsigargin (1 nM) was added to cuvette in calcium-free HBSS/0.5 mM EGTA buffer. Wavelength of excitation was alternately set to 340 and 380 nm, and emission fluorescence was recorded at 515 nm. The intracellular content of free calcium is directly correlated with the fluorescence intensity ratio of 340/380 nm (F340/F380) [19]. The results were expressed as the delta (Δ) of the Thapsigargin-induced increase in the basal fluorescence ratio (F340/F380 nm).

### 4.7. IL-6 Measurement

IL-6 levels in the supernatant of both cell lines were determined using an Enzyme-Linked Immuno Sorbent Assay (ELISA). Both cells were seeded in 96-well plates (5.0 × 10^4^ cells/well), left to attach for 24 h, and processed as previously described. ELISA was performed using a commercially available kit, following the producer’s instructions. Cytokine release was expressed as pg/mL.

### 4.8. Detection of Nitric Oxide Levels

Nitric Oxide (NO) levels were assessed in both cell lines (8 × 10^4^ cells/well), which were seeded in 6-well plates and processed as indicated above. After being treated, the cells were then harvested, flushed with PBS twice, and then incubated in PBS with 4,5-diaminofluorescein diacetate (DAF-2DA; 10 μM) probe for 1 h in the dark (37 °C). Fluorescence-activated cell sorting (FACSscan; Becton-Dickinson, Franklin Lakes, NJ, USA) was then used to measure the fluorescence of the cells, and Cell Quest software (version 4.1) was used to interpret the results.

### 4.9. Statistical Analysis

Analysis of data and statistical evaluations were performed using the commercially available program GraphPad Prism8 (GraphPad Software Inc., San Diego, CA, USA). The presented data represents the mean ± standard error of a minimum of three separate, technically duplicate studies. Non-parametric Mann–Whitney U method was used to collect statistical data between the experimental points. Differences were deemed significant if *p*-values were within the range of less than 0.01 to 0.05.

## Data Availability

The original contributions presented in this study are included in the article. Further inquiries can be directed to the corresponding author.
